# Prevalence of primary and secondary hypertension among hospitalized patients with cancer in the United States

**DOI:** 10.1177/17423953231196613

**Published:** 2023-08-22

**Authors:** Chanhyun Park, Sola Han, Kathryn P Litten, Sanica Mehta, Boon Peng Ng

**Affiliations:** 1 Health Outcomes Division, 15528 The University of Texas at Austin College of Pharmacy, Austin, TX, USA; 2 Pharmacy Practice Division, 15528 The University of Texas at Austin College of Pharmacy, Austin, TX, USA; 3 118710 The University of Texas at Austin College of Natural Sciences, Austin, TX, USA; 4 College of Nursing, 6243 University of Central Florida, Orlando, FL, USA; 5 Disability, Aging, and Technology Cluster, 6243 University of Central Florida, Orlando, FL, USA

**Keywords:** Hypertension, primary hypertension, secondary hypertension, cancer, hospitalization

## Abstract

**Background:**

Hypertension is the most common comorbidity in patients with cancer. We aimed to estimate the prevalence of hypertension by demographic characteristics and cancer type among hospitalized patients with cancer.

**Methods:**

Hospitalized cancer patients were included using 2016–2018 National Inpatient Sample data. The independent variable was the presence of hypertension, which was further classified as primary, secondary, and other hypertension. Patient characteristics were grouped by age, sex, race/ethnicity, and the 12 most common cancer types. Multinomial logistic regression was used.

**Results:**

Among 638,670 hospitalized patients with cancer, 56.8% had hypertension. The predicted percentages of having any hypertension were higher with age, male gender, and black race. The predicted percentages of any hypertension were the highest in kidney cancer patients across all age and race/ethnicity groups. Uterine cancer was associated with the highest percentages of primary hypertension, followed by kidney cancer. Leukemia was associated with the highest percentages of secondary hypertension, followed by non-Hodgkin lymphoma.

**Discussion:**

Kidney cancer patients had the highest predicted percentage of hypertension overall, while uterine cancer and leukemia had the highest percentages of primary and secondary hypertension, respectively. This study provides evidence for identifying cancer patients who need more attention for the prevention and management of hypertension.

## Introduction

Hypertension is the most common comorbidity in patients with cancer in the United States.^1^ Both hypertension and cancer share risk factors (e.g. age) as the pathophysiology of hypertension and cancer are intertwined.^2^ Hypertension is a risk factor for certain cancers,^3^ and using antihypertensive medications are also associated with certain cancers (e.g. kidney cancer).^4^ Conversely, several cancer treatments, such as chemotherapy and radiotherapy, increase the likelihood of developing or worsening hypertension.^1^

Hypertension can be classified based on its cause. The majority of patients fall under the primary hypertension diagnosis with cases due to genetic or environmental causes, while secondary hypertension defines cases that have a remediable cause and applies to about 10% of hypertension diagnoses.^5^ Screening for secondary causes of hypertension should be completed upon diagnosis and periodically throughout.^5^ While the risks and the treatment are typically the same among primary and secondary hypertension, it is important to recognize that secondary hypertension is a risk of some cancer treatments, so those most at risk can be monitored and treated appropriately with additional monitoring as the offending agents are removed to adjust hypertensive treatment as needed. Little is known about the prevalence of primary and secondary hypertension among patients with different types of cancer.

Morbidity and mortality of both hypertension and cancer can differ substantially by patients’ demographics due to various health care disparities. Age is one of the shared risk factors for developing hypertension and cancer.^6,7^ Although the likelihood of developing cardiovascular disease (CVD) at lower blood pressure thresholds is higher in females than males,^8^ males are more likely to develop both hypertension and cancer than females during their lifetime.^8,9^ Additionally, compared to White patients, the prevention, awareness, and management of hypertension are uniquely vulnerable for racial/ethnic minorities (i.e., Black, Asian, and Hispanic Americans) in the United States.^10^ Black Americans had the highest risk of cancer mortality, although White Americans are at the highest cancer incidence rate.^11^ Thus, despite the difference in the incidence of hypertension and cancer by demographic characteristics, no evidence is available about the prevalence of different types of hypertension stratified by age group, sex, and race/ethnicity among patients with cancer.

Hospital admission is often an inevitable process of cancer treatment.^12^ A multinational observational study found that extended hospital length of stay (LOS) was associated with certain combinations of comorbidities.^13^ Yet, no study examines whether having different types of comorbid hypertension would prolong the LOS in patients with cancer. Therefore, the primary objective of this study was to estimate the prevalence of primary, secondary, and other hypertension by demographic characteristics and cancer type among hospitalized patients with cancer in the United States. In addition, the secondary objective was to predict if the hospital LOS may be attributed to primary, secondary, and other hypertension in this population.

## Methods

### Data source

This study used the 2017–2018 National Inpatient Sample (NIS) database that is part of the Healthcare Cost and Utilization Project. NIS contains more than 7 million hospital discharges each year (a weighted estimate of approximately 35 million hospitalizations nationally).^14^

### Study population

**Figure 1.**
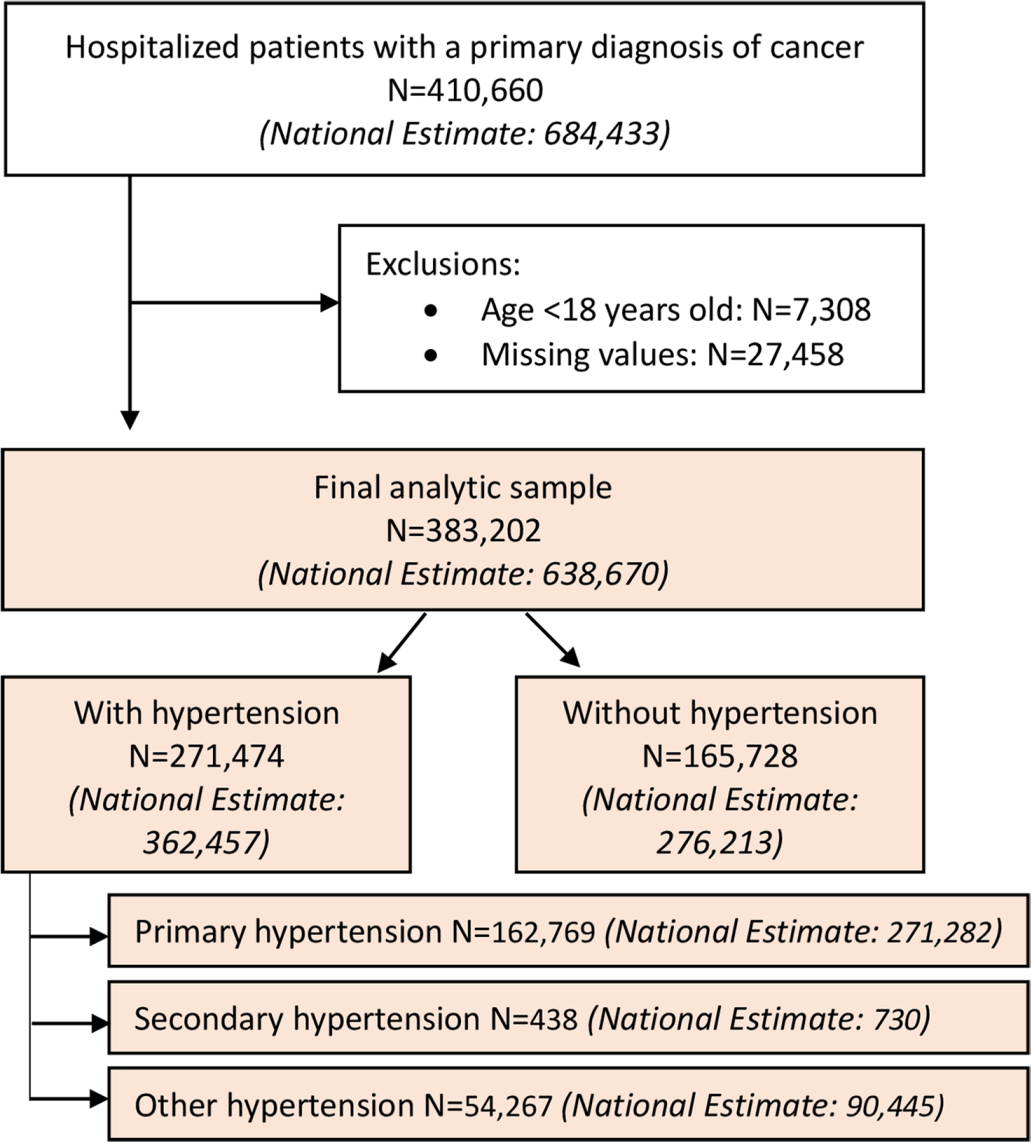
Patient selection process.

Patients were included if they were hospitalized with a primary diagnosis code for any cancer (International Classification of Diseases, Tenth Revision, Clinical Modification (ICD-10-CM) = C00-C96) (unweighted *n* = 410,660, national estimate with weights = 684,433). We excluded patients if they were aged less than 18 years (*n* = 7308) or had any missing values for patient/hospital characteristics (*n* = 27,458). Finally, a total of 383,202 hospitalized patients with cancer were included in this analysis (national estimate with weights = 638,670) (Figure 1).

### Variables

*Hypertension:* Our primary interest was the presence of comorbid hypertension. Comorbid hypertension during hospitalization was identified using ICD-10 diagnosis codes. Hypertension was further classified as primary hypertension (ICD-10-CM = I10), secondary (ICD-10-CM = I15), and other (hypertensive heart and/or renal disease and hypertensive crisis) (ICD-10-CM = I11, I12, I13, I16) hypertension.

*LOS:* Hospital LOS was defined as the days of a single episode of hospitalization (unit: days).

*Patient Characteristics:* We included the following demographic characteristics as covariates: age group (18–54, 55–64, 65–74, and 75+ years), sex (male and female), race/ethnicity (white, black, Hispanic, Asian, and others), household income, and primary payer. In addition, we included the following clinical information: Elixhauser index, comorbid CVD, (i.e. atrial fibrillation, coronary artery disease, cardiomegaly, cardiomyopathy, heart failure, peripheral artery disease, and stroke (Supplementary Table S1)), cancer treatments (i.e. cancer surgery, radiation, and chemotherapy).^15^ According to the Surveillance, Epidemiology, and End Results Program's statistics, we classified the top 12 common cancer of breast, prostate, lung/bronchus, colon/rectum, melanoma, urinary bladder, non-Hodgkin lymphoma, kidney/renal pelvis, corpus uteri, leukemia, pancreas, thyroid (Supplementary Table S1).^16^ Cancer patients without top 12 common cancer diagnoses were defined as other cancer patients.

*Hospital characteristics:* We included the following hospital characteristics as covariates: bed size (small, medium, and large), region of the hospital (Northeast, Midwest, South, West), and location/teaching status of the hospital (rural, urban: nonteaching, and urban: teaching).

### Statistical analysis

All analyses considered appropriate sampling weights and design effects of the national sample. The χ2 tests and *t*-test were used to examine the differences in the prevalence of hypertension by the type of cancer for all covariates. A multinomial logistic regression model was used to estimate the odds of having different types of hypertension by cancer patients’ clinical and demographic characteristics and hospital characteristics. A negative binomial regression model was used to assess the association of hypertension with LOS, controlling for clinical and demographic characteristics of cancer patients (e.g. comorbidities and cancer types) as well as hospital characteristics. We performed statistical analyses using SAS Version 9.4 and Stata/MP Version 17.

## Results

### Patient characteristics

**Figure 2.**
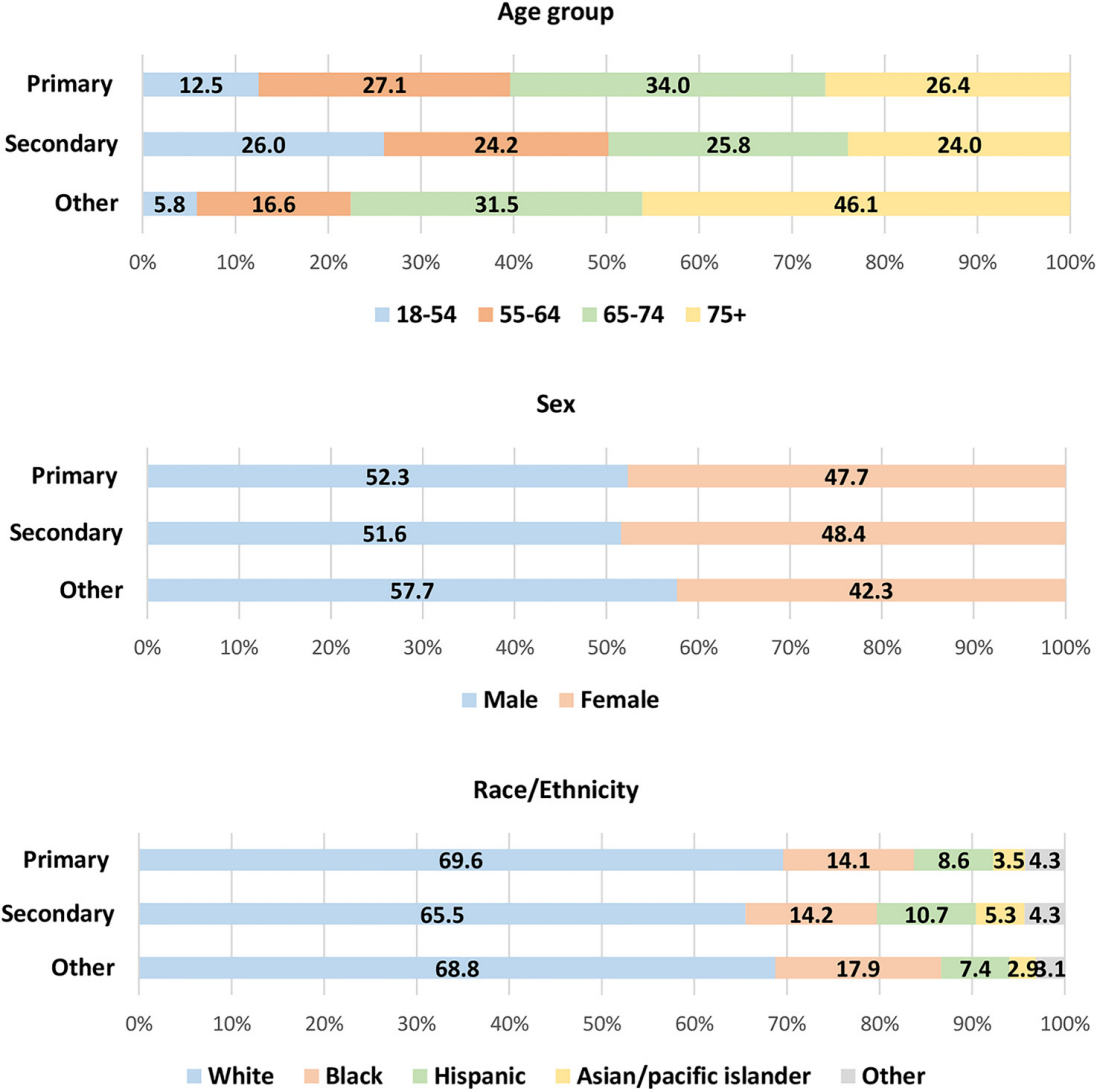
Percentage of patients by age, sex, and race/ethnicity for primary, secondary, and other hypertension (primary hypertension: unweighted *n* =  162,769, weighted *n* =  271,282; secondary: unweighted *n* = 438, weighted *n* = 730; other hypertension: unweighted *n* =  54,267, weighted *n* =  90,445).

Table 1 shows the patient and hospital characteristics for hospitalization with cancer by the presence of hypertension in the United States (weighted *n* = 638,670). Among them, 56.8% (weighted *n* = 362,457) had comorbid hypertension during hospitalization. The associations between the presence of hypertension and patient characteristics (e.g. age, sex, race/ethnicity, income, insurance, comorbidities) and hospital characteristics (e.g. bed size, region, location/teaching status) were found to be statistically significant. Among patients with comorbid hypertension, most of them had primary hypertension (weighted *n* = 271,282), followed by other hypertension (weighted *n* = 90,445) while secondary hypertension was rare (weighted *n* = 730) (Figure 1). Figure 2 depicts the distribution of patients by age, sex, and race/ethnicity for each primary, secondary, and other hypertension. Compared to the distribution of age group among patients with primary or other hypertension, percentages of patients with secondary hypertension were relatively evenly distributed among different age groups. Additionally, Supplementary Table S2 shows the full information about patient and hospital characteristics for hospitalization with cancer by type of hypertension.

**Table 1.** Patient and hospital characteristics for hospitalization with cancer by hypertension status in the United States.

	Hypertension		Nonhypertension		Prevalence^a^	*p*
	Unweighted *n* 217,474		Unweighted *n* 165,728		56.8	
	Weighted *n* 362,457		Weighted *n* 276,213			
	*n*	(%)	*n*	(%)		
Patient characteristics						
Age, mean (SE)	68.5	(0.1)	59.4	(0.1)		<0.001
Age groups						
18–54	39,413	(10.9)	90,543	(32.8)	30.3	<0.001
55–64	88,643	(24.5)	80,667	(29.2)	52.4	
65–74	120,905	(33.4)	66,440	(24.1)	64.5	
75+	113,495	(31.3)	38,563	(14.0)	74.6	
Sex						
Male	194,427	(53.6)	137,888	(49.9)	58.5	<0.001
Female	168,030	(46.4)	138,325	(50.1)	54.9	
Race						
White	251,445	(69.4)	195,748	(70.9)	56.2	<0.001
Black	54,612	(15.1)	26,705	(9.7)	67.2	
Hispanic	29,993	(8.3)	28,582	(10.4)	51.2	
Asian/Pacific Islander	12,002	(3.3)	11,315	(4.1)	51.5	
Other	14,405	(4.0)	13,863	(5.0)	51.0	
Income						
0–25th	99,058	(27.3)	65,838	(23.8)	60.1	<.001
26–50th	93,765	(25.9)	68,667	(24.9)	57.7	
51–75th	88,112	(24.3)	69,250	(25.1)	56.0	
76–100th	81,522	(22.5)	72,458	(26.2)	52.9	
Insurance						
Medicare	225,082	(62.1)	103,545	(37.5)	68.5	<.001
Medicaid	30,330	(8.4)	39,105	(14.2)	43.7	
Private insurance	91,935	(25.4)	114,852	(41.6)	44.5	
Other	15,110	(4.2)	18,712	(6.8)	44.7	
Elixhauser, mean (SE)	5.9	(0.1)	7.0	(0.1)		<.001
Comorbidities						
Atrial fibrillation	54,210	(15.0)	17,040	(6.2)	76.1	<.001
Coronary artery disease	78,215	(21.6)	16,935	(6.1)	82.2	<.001
Cardiomegaly	928	(0.3)	458	(0.2)	67.0	<.001
Cardiomyopathy	7080	(2.0)	2023	(0.7)	77.8	<.001
Heart failure	40,407	(11.2)	7367	(2.7)	84.6	<.001
Peripheral artery disease	15,298	(4.2)	4088	(1.5)	78.9	<.001
Stroke	15,338	(4.2)	5948	(2.2)	72.1	<.001
Cancer treatments						
Cancer surgery	101,302	(28.0)	76,513	(27.7)	57.0	.140
Radiation	6250	(1.7)	5103	(1.9)	55.1	.007
Chemotherapy	13,568	(3.7)	14,247	(5.2)	48.8	<.001
Cancer types						
Breast	9855	(2.7)	14,035	(5.1)	41.3	<.001
Prostate	22,622	(6.2)	18,813	(6.8)	54.6	<.001
Lung and bronchus	48,300	(13.3)	28,430	(10.3)	63.0	<.001
Colon and rectum	46,342	(12.8)	34,848	(12.6)	57.1	.148
Melanoma of the skin	710	(0.2)	555	(0.2)	56.1	.720
Urinary bladder	10,758	(3.0)	5460	(2.0)	66.3	<.001
Non-Hodgkin lymphoma	12,398	(3.4)	10,443	(3.8)	54.3	<.001
Kidney and renal pelvis	20,378	(5.6)	9927	(3.6)	67.2	<.001
Corpus uteri	7713	(2.1)	4598	(1.7)	62.7	<.001
Leukemia	12,262	(3.4)	11,757	(4.3)	51.1	<.001
Pancreas	15,002	(4.1)	8647	(3.1)	63.4	<.001
Thyroid	2557	(0.7)	3383	(1.2)	43.0	<.001
Other	153,560	(42.4)	125,317	(45.4)	55.1	<.001
Hospital characteristics						
Bed size						
Small	50,172	(13.8)	37,142	(13.5)	57.5	<.001
Medium	93,550	(25.8)	67,015	(24.3)	58.3	
Large	218,735	(60.4)	172,057	(62.3)	56.0	
Region						
Northeast	77,800	(21.5)	61,572	(22.3)	55.8	<.001
Midwest	79,408	(21.9)	54,685	(19.8)	59.2	
South	142,228	(39.2)	100,752	(36.5)	58.5	
West	63,020	(17.4)	59,205	(21.4)	51.6	
Location/teaching status						
Rural	15,542	(4.3)	11,138	(4.0)	58.3	<.001
Urban nonteaching	57,012	(15.7)	39,618	(14.3)	59.0	
Urban teaching	289,903	(80.0)	225,457	(81.6)	56.3	

^a^ Prevalence was obtained as the percentage of patients with hypertension per each category.

### Prevalence of hypertension

**Figure 3.**
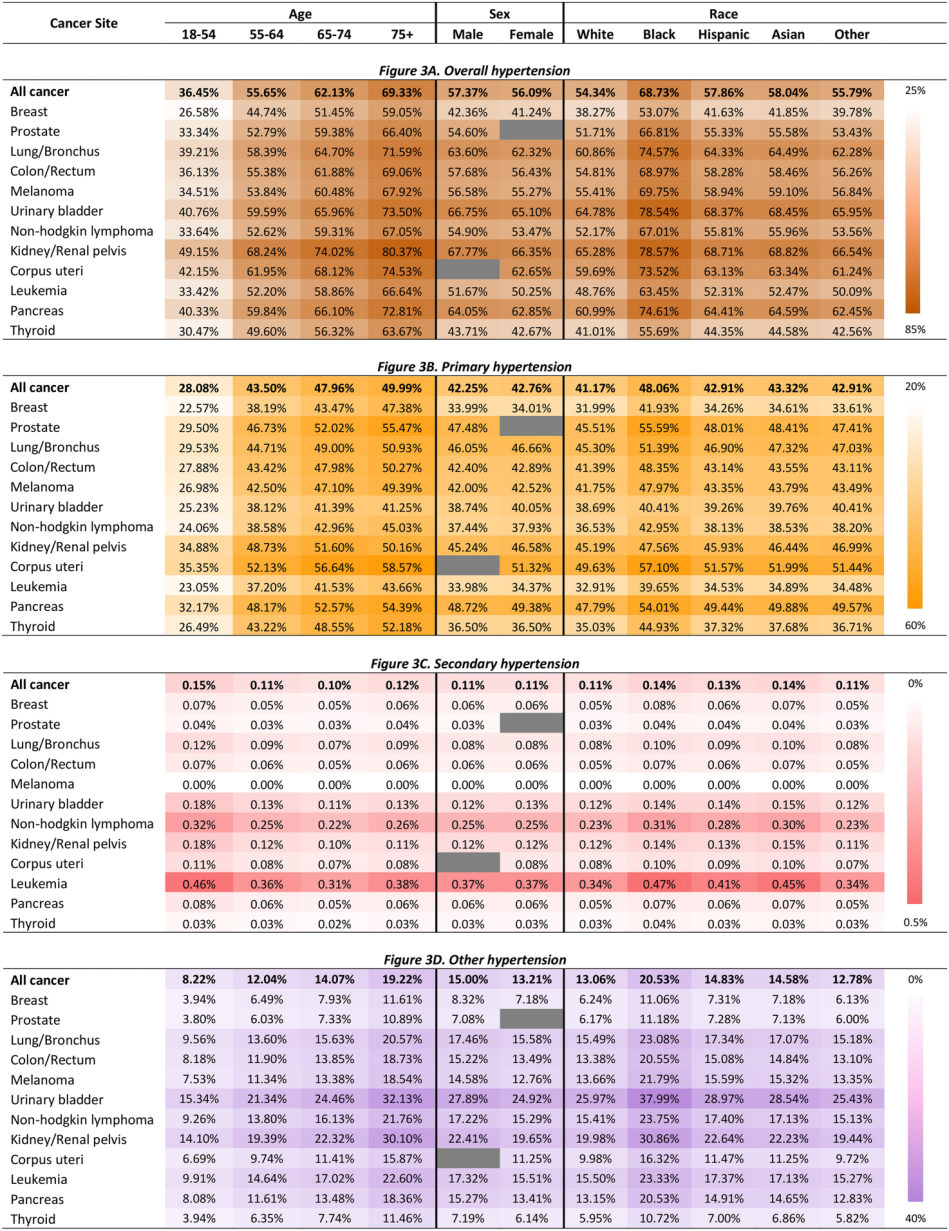
Predicted probabilities of having comorbid hypertension by patient characteristics and cancer type in hospitalized patients with cancer in the United States.

Figure 3 shows the predicted probabilities of having each type of hypertension by age group, sex, race/ethnicity, and cancer type in hospitalized patients with cancer, controlling for covariate. The results were from a multinomial logistic regression model. The information on relevant relative risk ratios is available in Supplementary Table S3, and standard errors in Supplementary Table S4.

For overall hypertension, compared to patients aged 18–54 years (36.45%), the predicted percentages of having any hypertension diagnosis were higher in those aged 55–64 (55.65%), those aged 65–74 (62.13%) and those aged 75+ (69.33%). Regarding age group and the type of hypertension, the predicted percentages of having comorbid primary hypertension or other hypertension were higher in patients aged 55–64 (43.50% and 12.04%), 65–74 (47.96% and 14.07%), 75+ (49.99% and 19.22%), compared to patients aged 18–54 years (28.08% and 8.22%). However, the predicted percentage of having comorbid secondary hypertension was the highest in patients aged 18–54 years (0.15%) than those aged in those aged 55–64 (0.11%), 65–74 (0.10%), and 75+ (0.12%).

The predicted percentage of having hypertension was higher in male patients (57.37%) than in female (56.09%). Compared to white patients (54.34%), the predicted percentages of having hypertension were higher in Black (68.73%), Hispanic (57.86%), and Asian (58.04%) patients.

Among the different types of cancer, the predicted percentages of hypertension were the highest in kidney cancer patients across all ages (range: 49.15% for 18–54 years–80.37% for 75+ years) and race/ethnicity groups (range: 65.28% for White–max: 78.57% for Black), followed by uterine and bladder cancers. Regarding cancer site and the type of hypertension, uterine cancer was associated with the highest predictive percentages of primary hypertension, followed by kidney; leukemia was associated with the highest predictive percentages of secondary hypertension, followed by non-Hodgkin lymphoma; bladder was associated with the highest predictive percentages of other hypertension, followed by kidney.

### Hypertension and LOS

**Figure 4.**
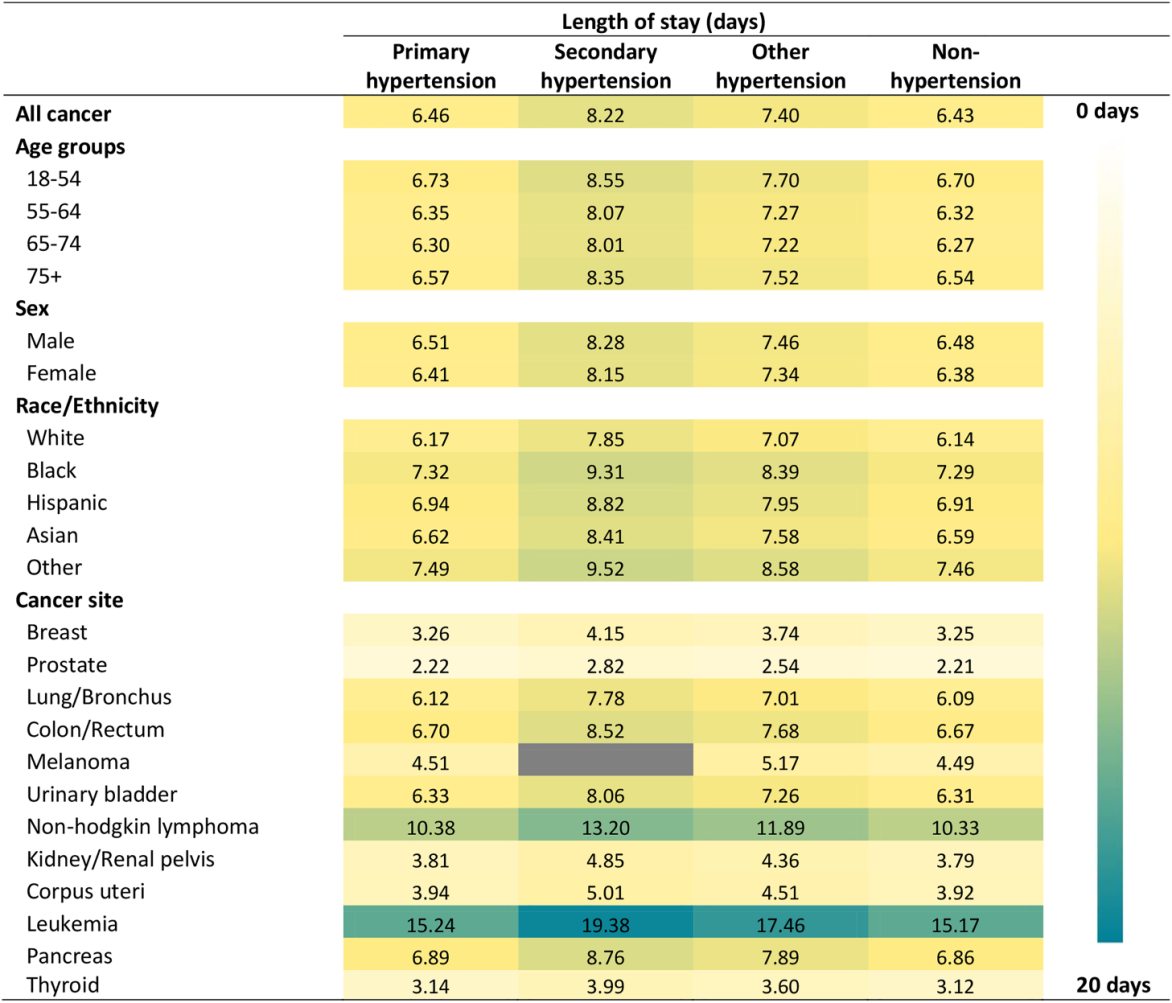
Estimated hospital length of stay by patient characteristics and cancer type in hospitalized patients with cancer stratified by hypertension type.

Figure 4 shows the predicted hospital LOS stratified by the type of hypertension by age group, sex, race/ethnicity, and cancer type in hospitalized patients with cancer, controlling for covariates. The results were from a negative binomial regression model. The information on relevant incidence rate ratio (IRR) is available in Supplementary Table S5 and standard errors in Supplementary Table S6. Compared to not having hypertension, having comorbid primary hypertension was not associated with an increase in hospital LOS among cancer patients (IRR = 1.00, 95% CI = 1.00, 1.01, *p* = .316). However, having comorbid secondary and other hypertension was associated with a 28% and 15% increase in hospital LOS (IRR = 1.28, 95% CI = 1.17, 1.39, *p* < .001, and IRR = 1.15, 95% CI = 1.13, 1.17, *p* < .001). Among hospitalized patients with cancer, the predicted mean LOS were 6.46 days (SE = 0.03) in those with primary hypertension, 8.22 days (SE = 0.36) in those with secondary hypertension, 7.40 days (SE = 0.05) in those with other hypertension, and 6.43 days (SE = 0.04) in those without hypertension.

## Discussion

Hypertension is the most common comorbidity in patients with cancer caused by shared risk factors, cancer treatment-induced cardiovascular complications.^1,2^ The morbidity and mortality of both hypertension and cancer are all influenced by age and race/ethnicity.^1,10,17^ Nevertheless, little is known about the prevalence of different types of hypertension stratified by cancer type for each age group, sex, and race/ethnicity. This study filled this gap by estimating the prevalence of different types of hypertension by demographic characteristics and cancer type among hospitalized patients with cancer in the United States. Overall, we found that approximately 56.8% of hospitalizations of cancer patients also had hypertension, and the predicted percentages of any hypertension were the highest in kidney cancer patients across all age and race/ethnicity group followed by uterine and bladder cancers, but leukemia was associated with the highest predictive percentages of secondary hypertension.

Age and race/ethnicity are the factors associated with the presence of comorbid hypertension among hospitalized patients with cancer. Specifically, we found that older age was associated with primary and other hypertension–hypertensive heart or renal disease–but not secondary hypertension, among hospitalized patients with cancer. Similar to our finding for primary and other hypertension, prior studies have found similar patterns of hypertension in cancer patient populations with stronger associations in elderly patients.^18,19^ Additionally, the literature has shown that older age is a risk factor for hypertension upon treatment by certain chemotherapeutic agents.^20,21^ However, older age was not directly associated with the incidence of secondary hypertension among hospitalized patients with cancer, indicating age might not an independent risk factor for developing secondary hypertension. This may indicate that secondary hypertension in this population may be more due to drug-induced causes rather than other comorbidities generally associated with secondary hypertension which often do correlate with age.^5^

Race/ethnicity was also a factor in predicting the presence of comorbid hypertension among hospitalized patients with cancer. We found that compared to white patients, black patients had higher predicted percentages of having hypertension. Black patients had about a one-seventh higher expected rate than white patients. This finding is in line with previous studies that found the prevalence of hypertension in black cancer patients was almost twice the prevalence in white patients.^19,22^ Since hypertension is associated with a one-fourth increased risk of mortality from cancer, it is crucial to address this discrepancy in hypertension.^18^

We found that the prevalence of different types of hypertension varied by the type of cancer. First, we found that kidney cancer was associated with the highest predictive percentages of all types of hypertension. The pervasiveness of hypertension in kidney cancer patients across all ages and races was nearly two-thirds. This finding is congruent with previous studies that consistently found positive associations with kidney cancer indicating that a hypertension prevalence of three-fourths among kidney cancer patients, while associations between hypertension and other malignancies can vary.^3,7,23^ There are correlations between hypertension and kidney cancer. Hypertension is known as an independent risk factor for the development of kidney cancer.^24^ On the other hand, kidney cancer patients are at a higher risk of developing hypertension with the use of tyrosine-kinase inhibitors (TKI), which have been considered first-line treatment for metastatic renal cell cancer, and hypertension is one of the common adverse events of TKI.^25^ Therefore, promoting adequate prevention and management of hypertension may enhance health outcomes for hospitalized patients with kidney cancer.

Second, concerning specific types of hypertension, we found that uterine cancer, followed by kidney cancer, was associated with the highest predictive percentages of primary hypertension. The literature has conflicting conclusions regarding the identification of an association between uterine cancer and hypertension. Hypertension has been associated with an increased risk of uterine cancer with a reported prevalence of around one-fourth of patients, but the results have not been consistent.^7,26^ A recent meta-analysis concluded that the relative risk of endometrial cancer may increase by 61% in women with hypertension.^27^ However, they suggested further studies to clarify potential effect modification, the causality of this association, and the potential underlying mechanisms.

Third, in this study, leukemia was associated with the highest predictive percentage of secondary hypertension, followed by non-Hodgkin lymphoma. This could be due to the fact that patients with lymphoma and leukemia are treated with alkylating agents, which are known to cause hypertension.^2^ Other treatments, such as corticosteroids and TKIs are other common treatments with hypertension as a side effect.^2^ Previous studies have found that for those with lymphoma and leukemia, hypertension was the strongest predictor of heart failure due to treatment by certain chemotherapeutic treatments.^3^

Finally, bladder cancer was associated with the highest predictive percentages of other hypertension, including hypertensive heart and/or renal disease. A recent population-based cohort study found a positive association between essential/primary hypertension and subsequent risk of developing bladder cancer.^28^ In addition, another cohort study found that systolic blood pressure was positively associated with bladder cancer-specific mortality.^29^ Therefore, health outcomes for hospitalized patients with bladder cancer may be improved by promoting effective hypertension prevention and management strategy.

We quantified the increased LOS due to hypertension among hospitalized patients with cancer. Primary hypertension was not associated with an increase in hospital LOS, while comorbid secondary hypertension was associated with an increase in hospital LOS. The increase in secondary hypertension may be due to starting new cancer therapies or high-dose steroids in the hospital leading to acute increases in blood pressure that must be treated. The differences in LOS can have high impact outcomes on both the patient and healthcare system financially, operationally, and clinically.

There are several potential limitations in this study. First, we were unable to obtain information on the several clinical characteristics of the included patients (e.g. cancer stage,) due to the limitation of the variables in the NIS data. The lack of data on the cancer stage may potentially impact the outcomes of our study. Second, patients may have been counted more than once if they were hospitalized multiple times during the study time frame since the NIS data captured hospitalization events. Third**,** we were unable to establish a causal relationship between hypertension and LOS due to the characteristics of cross-sectional data. In addition, the diagnosis category of other hypertension may have included those with primary or secondary hypertension that led to complications or were related to other comorbidities. Those categorized with primary or secondary may have also had these complications which were not specifically identified via the ICD 10 code pulled for this study.

Nevertheless, this study has several strengths. First, this is the first study about estimating the prevalence of different types of comorbid hypertension among hospitalized cancer patients stratified by patient demographics and cancer type at the U.S. population level. In addition, this study provides evidence for improving care in patients with cancer and hypertension, given the high rates identified. This promotes a call to action for the development of hypertension prevention and treatment strategies to avoid poor patient outcomes and added healthcare costs.

Several statements from cardio-oncology societies have been made in recent years highlighting the need to identify and control cardiotoxicities associated with antineoplastic therapy, including hypertension.^30–33^ An algorithm for screening, monitoring, and treatment of blood pressure in patients with cancer receiving antineoplastic therapy known to be associated with hypertension.^34^ Important aspects include risk factor screening, early detection and treatment, and employing a multidisciplinary approach. Providing patients with home blood pressure monitors upon diagnosis of hypertension or cancer may aid in this early detection and ultimate treatment titration.^5^ More frequent monitoring throughout and even following cancer treatment may be necessary for this population.

One recommendation has been to optimize blood pressure control before beginning cancer treatment, particularly for those at risk for worsening hypertension or with higher cardiovascular risk.^34^ When possible, the hypertensive profile of the cancer medication should be considered prior to initiation, though this may not always be avoidable. Cancer regimens should also be considered when optimizing antihypertensive treatment. Mechanisms of the cause of hypertension differ among cancer drug classes. First-line agents for the treatment of hypertension may target or inhibit the cause of blood pressure increase for more effect.^2,30–32,34^

This study may help identify and promote prevention or treatment protocols for those at higher risk for hypertension such as those who are older adults, black, or who have cancers with higher rates identified such as kidney or uterine cancer. Those with high rates of cancer-related to secondary hypertension likely due to prohypertensive medications, such as leukemia should be counseled and monitored prior to treatment with these agents. Monitoring and appropriate treatment in the outpatient setting may help prevent hospitalizations, and complications.^5^ Preventing complications and uncontrolled secondary hypertension may decrease hospital LOS.

## Conclusions

In conclusion, hypertension is highly prevalent among hospitalized cancer patients, especially kidney cancer. This study emphasizes the need to control hypertension in all cancer patients, but highlights specific patient populations (e.g. kidney, bladder, and uterine cancers and blood cancers) to target as highest risk in order to develop treatment and prevention protocols to focus impact.

## Supplemental Material

sj-docx-1-chi-10.1177_17423953231196613 - Supplemental material for Prevalence of primary and secondary hypertension among hospitalized patients with cancer in the United States Supplemental material, sj-docx-1-chi-10.1177_17423953231196613 for Prevalence of primary and secondary hypertension among hospitalized patients with cancer in the United States by Chanhyun Park, Sola Han, Kathryn P Litten, Sanica Mehta and Boon Peng Ng in Chronic Illness
