# Supplementary material for: Prevalence of primary and secondary hypertension among hospitalized patients with cancer in the United States
Source: Chronic Illn. 2023 Aug 22;21(1):42–55. doi: 10.1177/17423953231196613 (PMC11969873; doi:10.1177/17423953231196613)
Supplement: sj-docx-1-chi-10.1177_17423953231196613 - Supplemental material for Prevalence of primary and secondary hypertension among hospitalized patients with cancer in the United States [file sj-docx-1-chi-10.1177_17423953231196613.docx]

**Supplementary Appendix**

**Supplementary Table S1. International Classification of Diseases, Tenth Revision, Clinical Modification (ICD-10-CM)**

| Cardiovascular Disease |
| --- |
| atrial fibrillation (I48) |
| coronary artery disease (I20-I25, I252) |
| cardiomegaly (I517) |
| cardiomyopathy (I43, I427, I429, I420, I425) |
| heart failure (I50) |
| peripheral artery disease (I70, I74, I739) |
| stroke (I60-I63, I65-I66, I69, I672, I679, I6781-I6782) |
|  |
| Cancer |
| breast (ICD-10-CM = C50), |
| prostate (ICD-10-CM = C61), |
| lung/bronchus (ICD-10-CM = C34), |
| colon/rectum (ICD-10-CM = C18-C21, C260), |
| melanoma (ICD-10-CM = C43), |
| urinary bladder (ICD-10-CM = C67) |
| non-Hodgkin lymphoma (ICD-10-CM = C82-C86, C963) |
| kidney/renal pelvis (ICD-10-CM = C64-C65) |
| corpus uteri (ICD-10-CM = C54) |
| leukemia (ICD-10-CM = C91-C93, C95, C940-C945, C947) |
| pancreas (ICD-10-CM = C25) |
| thyroid (ICD-10-CM = C7) |

**Supplementary Table S2. Patient and hospital characteristics for hospitalization with cancer by hypertension type in the U.S. (n=271,474, weighted n=362,457)**

|  | **Primary hypertension** | | | **Secondary hypertension** | | **Other hypertension** | | ***p*** |
| --- | --- | --- | --- | --- | --- | --- | --- | --- |
|  | Unweighted N | | 162769 | Unweighted N | 438 | Unweighted N | 54267 |  |
|  | Weighted N | | 271282 | Weighted N | 730 | Weighted N | 90445 |  |
|  | n | | Weighted % | n | Weighted % | n | Weighted % |  |
| **Patient characteristics** |  | |  |  |  |  |  |  |
| **Age, mean (SE)** | 67.2 | | (0.1) | 62.2 | (0.8) | 72.6 | (0.1) | <0.001 |
| **Age groups** |  | |  |  |  |  |  |  |
| 18-54 | 33945 | | (12.5) | 190 | (26.0) | 5278 | (5.8) | <0.001 |
| 55-64 | 73463 | | (27.1) | 177 | (24.2) | 15003 | (16.6) |  |
| 65-74 | 92270 | | (34.0) | 188 | (25.8) | 28447 | (31.5) |  |
| 75+ | 71603 | | (26.4) | 175 | (24.0) | 41717 | (46.1) |  |
| **Sex** |  | |  |  |  |  |  |  |
| Male | 141878 | | (52.3) | 377 | (51.6) | 52172 | (57.7) | <0.001 |
| Female | 129403 | | (47.7) | 353 | (48.4) | 38273 | (42.3) |  |
| **Race/Ethnicity** |  | |  |  |  |  |  |  |
| White | 188787 | | (69.6) | 478 | (65.5) | 62180 | (68.8) | <0.001 |
| Black | 38322 | | (14.1) | 103 | (14.2) | 16187 | (17.9) |  |
| Hispanic | 23227 | | (8.6) | 78 | (10.7) | 6688 | (7.4) |  |
| Asian/pacific islander | 9373 | | (3.5) | 38 | (5.3) | 2590 | (2.9) |  |
| Other | 11573 | | (4.3) | 32 | (4.3) | 2800 | (3.1) |  |
| **Income** |  | |  |  |  |  |  |  |
| 0-25th | 72967 | | (26.9) | 163 | (22.4) | 25928 | (28.7) | <0.001 |
| 26-50th | 70148 | | (25.9) | 182 | (24.9) | 23435 | (25.9) |  |
| 51-75th | 66093 | | (24.4) | 187 | (25.6) | 21832 | (24.1) |  |
| 76-100th | 62073 | | (22.9) | 198 | (27.2) | 19250 | (21.3) |  |
| **Insurance** |  | |  |  |  |  |  |  |
| Medicare | 156353 | | (57.6) | 370 | (50.7) | 68358 | (75.6) | <0.001 |
| Medicaid | 24602 | | (9.1) | 85 | (11.6) | 5643 | (6.2) |  |
| Private insurance | 78048 | | (28.8) | 255 | (34.9) | 13632 | (15.1) |  |
| Other | 12278 | | (4.5) | 20 | (2.7) | 2812 | (3.1) |  |
| **Elixhauser index, mean (SE)** | | 5.77 | (0.1) | 6.66 | (0.5) | 6.43 | (0.1) |  |
| **Comorbidities** |  | |  |  |  |  |  |  |
| Atrial fibrillation | 29957 | | (11.0) | 72 | (9.8) | 24182 | (26.7) | <0.001 |
| Coronary artery disease | 45052 | | (16.6) | 110 | (15.1) | 33053 | (36.6) | <0.001 |
| Cardiomegaly or  Cardiomyopathy | 2447 | | (0.9) | 18 | (2.5) | 5543 | (6.1) | <0.001 |
| Heart failure | 2058 | | (0.8) | 50 | (6.9) | 38298 | (42.3) | <0.001 |
| Peripheral artery disease | 9092 | | (3.4) | 45 | (6.2) | 6162 | (6.8) | <0.001 |
| Stroke | 10170 | | (3.8) | 52 | (7.1) | 5117 | (5.7) | <0.001 |
| **Cancer treatments** |  | |  |  |  |  |  |  |
| Cancer surgery | 83003 | | (30.6) | 125 | (17.1) | 18173 | (20.1) | <0.001 |
| Radiation | 4768 | | (1.8) | 33 | (4.6) | 1448 | (1.6) | <0.001 |
| Chemotherapy | 10057 | | (3.7) | 128 | (17.6) | 3383 | (3.7) | <0.001 |
| **Cancer types** |  | |  |  |  |  |  |  |
| Breast | 8123 | | (3.0) | 13 | (1.8) | 1718 | (1.9) | <0.001 |
| Prostate | 19675 | | (7.3) | 13 | (1.8) | 2933 | (3.2) | <0.001 |
| Lung and broncus | 35545 | | (13.1) | 63 | (8.7) | 12692 | (14.0) | <0.001 |
| Colon and rectum | 34623 | | (12.8) | 45 | (6.2) | 11673 | (12.9) | <0.001 |
| Urinary bladder | 6333 | | (2.3) | 20 | (2.7) | 4405 | (4.9) | <0.001 |
| Non-hodgkin lymphoma | 8600 | | (3.2) | 57 | (7.8) | 3742 | (4.1) | <0.001 |
| Kidney and renal pelvis | 13862 | | (5.1) | 37 | (5.0) | 6480 | (7.2) | <0.001 |
| Leukemia | 8200 | | (3.0) | 88 | (12.1) | 3973 | (4.4) | <0.001 |
| Pancreas | 11603 | | (4.3) | 13 | (1.8) | 3385 | (3.7) | <0.001 |
| Other* | 124716 | | (46.0) | 380 | (52.1) | 39443 | (43.6) | <0.001 |
| **Hospital characteristics** |  | |  |  |  |  |  |  |
| **Bed size** |  | |  |  |  |  |  |  |
| Small | 36947 | | (13.6) | 78 | (10.7) | 13147 | (14.5) | <0.001 |
| Medium | 68953 | | (25.4) | 170 | (23.3) | 24427 | (27.0) |  |
| Large | 165382 | | (61.0) | 482 | (66.0) | 52872 | (58.5) |  |
| **Region** |  | |  |  |  |  |  |  |
| Northeast | 59292 | | (21.9) | 92 | (12.6) | 18417 | (20.4) | <0.001 |
| Midwest | 57430 | | (21.2) | 217 | (29.7) | 21762 | (24.1) |  |
| South | 107637 | | (39.7) | 227 | (31.1) | 34365 | (38.0) |  |
| West | 46923 | | (17.3) | 195 | (26.7) | 15902 | (17.6) |  |
| **Location/teaching status** |  | |  |  |  |  |  |  |
| Rural | 11397 | | (4.2) | 12 | (1.6) | 4133 | (4.6) | <0.001 |
| Urban non-teaching | 41712 | | (15.4) | 102 | (13.9) | 15198 | (16.8) |  |
| Urban teaching | 218173 | | (80.4) | 617 | (84.5) | 71113 | (78.6) |  |

*Other included melanoma of the skin, corpus uteri, and thyroid.

**Supplementary Table S3. Adjusted relative risk ratios (RRR) of having primary, secondary, and other hypertension among hospitalized patients with cancer: results from multinomial logistic regression**

|  | ***Primary hypertension***  ***vs. Non-hypertension*** | | ***Secondary hypertension***  ***vs. Non-hypertension*** | | ***Other hypertension***  ***vs. Non-hypertension*** | | |
| --- | --- | --- | --- | --- | --- | --- | --- |
| **Variable** | **RRR (95% CI)^a^** | ***p*** | **RRR (95% CI)^a^** | ***p*** | **RRR (95% CI)^a^** | ***p*** | |
| **Patient Charateristics** |  |  |  |  |  |  | |
| **Age groups** |  |  |  |  |  |  | |
| 18-54 | Ref. |  | Ref. |  | Ref. |  | |
| 55-64 | 2.31 (2.26-2.36) | <0.001 | 1.12 (0.86-1.46) | 0.383 | 2.58 (2.46-2.70) | <0.001 | |
| 65-74 | 3.01 (2.93-3.11) | <0.001 | 1.17 (0.82-1.67) | 0.395 | 3.86 (3.64-4.08) | <0.001 | |
| 75+ | 3.93 (3.80-4.06) | <0.001 | 1.80 (1.22-2.67) | 0.003 | 7.66 (7.20-8.14) | <0.001 | |
| **Sex** |  |  |  |  |  |  | |
| Female | Ref. |  | Ref. |  | Ref. |  | |
| Male | 1.03 (1.01-1.04) | 0.002 | 1.04 (0.85-1.27) | 0.702 | 1.27 (1.23-1.31) | <0.001 | |
| **Race/Ethnicity** |  |  |  |  |  |  | |
| White | Ref. |  | Ref. |  | Ref. |  | |
| Black | 1.86 (1.81-1.90) | <0.001 | 2.10 (1.55-2.84) | <0.001 | 3.23 (3.11-3.36) | <0.001 | |
| Hispanic | 1.15 (1.12-1.19) | <0.001 | 1.32 (0.89-1.96) | 0.166 | 1.35 (1.29-1.42) | <0.001 | |
| Asian/pacific islander | 1.17 (1.11-1.23) | <0.001 | 1.45 (0.93-2.27) | 0.103 | 1.32 (1.22-1.44) | <0.001 | |
| Other | 1.08 (1.04-1.13) | <0.001 | 1.03 (0.55-1.92) | 0.931 | 1.01 (0.89-1.14) | 0.928 | |
| **Income** |  |  |  |  |  |  | |
| 0-25^th^ | Ref. |  | Ref. |  | Ref. |  | |
| 26-50^th^ | 0.96 (0.94-0.98) | 0.001 | 1.10 (0.81-1.48) | 0.537 | 0.98 (0.94-1.01) | 0.194 | |
| 51-75^th^ | 0.91 (0.89-0.93) | <0.001 | 1.10 (0.80-1.50) | 0.560 | 0.93 (0.89-0.97) | <0.001 | |
| 76-100^th^ | 0.84 (0.82-0.86) | <0.001 | 1.18 (0.87-1.61) | 0.288 | 0.83 (0.80-0.87) | <0.001 | |
| **Insurance** |  |  |  |  |  |  | |
| Medicare | Ref. |  | Ref. |  | Ref. |  | |
| Medicaid | 0.82 (0.79-0.84) | <0.001 | 0.68 (0.45-1.02) | 0.063 | 0.64 (0.61-0.68) | <0.001 | |
| Private insurance | 0.84 (0.82-0.86) | <0.001 | 0.82 (0.59-1.14) | 0.238 | 0.60 (0.57-0.63) | <0.001 | |
| Other | 0.72 (0.69-0.75) | <0.001 | 0.36 (0.20-0.68) | 0.001 | 0.53 (0.50-0.57) | <0.001 | |
| **Elixhauser Index** | 0.99 (0.99-0.99) | <0.001 | 1.00 (0.99-1.01) | 0.959 | 0.99 (0.99-0.99) | <0.001 | |
| **Comorbidities** |  |  |  |  |  |  | |
| Atrial fibrillation |  |  |  |  |  |  | |
| No | Ref. |  | Ref. |  | Ref. |  | |
| Yes | 1.35 (1.31-1.38) | <0.001 | 1.10 (0.79-1.52) | 0.587 | 1.63 (1.57-1.70) | <0.001 | |
| Coronary artery disease |  |  |  |  |  |  | |
| No | Ref. |  | Ref. |  | Ref. |  | |
| Yes | 2.34 (2.28-2.41) | <0.001 | 2.07 (1.56-2.76) | <0.001 | 3.22 (3.11-3.34) | <0.001 | |
| Cardiomegaly |  |  |  |  |  |  | |
| No | Ref. |  | Ref. |  | Ref. |  | |
| Yes | 0.86 (0.73-1.03) | 0.097 | 5.33 (2.23-12.72) | <0.001 | 2.84 (2.22-3.63) | <0.001 | |
| Cardiomyopathy |  |  |  |  |  |  | |
| No | Ref. |  | Ref. |  | Ref. |  | |
| Yes | 1.04 (0.95-1.14) | 0.356 | 0.98 (0.42-2.26) | 0.960 | 1.36 (1.24-1.50) | <0.001 | |
| Heart failure |  |  |  |  |  |  | |
| No | Ref. |  | Ref. |  | Ref. |  | |
| Yes | 0.18 (0.16-0.20) | <0.001 | 1.88 (1.27-2.80) | 0.002 | 13.26 (12.69-13.86) | | <0.001 |
| Peripheral artery disease |  |  |  |  |  |  | |
| No | Ref. |  | Ref. |  | Ref. |  | |
| Yes | 1.53 (1.44-1.61) | <0.001 | 2.92 (1.95-4.37) | <0.001 | 2.08 (1.95-2.23) | <0.001 | |
| Stroke |  |  |  |  |  |  | |
| No | Ref. |  | Ref. |  | Ref. |  | |
| Yes | 1.51 (1.44-1.58) | <0.001 | 2.55 (1.79-3.63) | <0.001 | 1.77 (1.66-1.89) | <0.001 | |
| **Cancer treatments** |  |  |  |  |  |  | |
| Cancer surgery |  |  |  |  |  |  | |
| No | Ref. |  | Ref. |  | Ref. |  | |
| Yes | 1.08 (1.06-1.10) | <0.001 | 1.00 (0.71-1.40) | 0.987 | 0.77 (0.74-0.80) | <0.001 | |
| Radiation |  |  |  |  |  |  | |
| No | Ref. |  | Ref. |  | Ref. |  | |
| Yes | 1.08 (1.02-1.14) | 0.007 | 2.13 (1.31-3.45) | 0.002 | 0.92 (0.83-1.02) | 0.096 | |
| Chemotherapy |  |  |  |  |  |  | |
| No | Ref. |  | Ref. |  | Ref. |  | |
| Yes | 0.98 (0.94-1.02) | 0.363 | 2.87 (2.10-3.91) | <0.001 | 1.02 (0.95-1.09) | 0.630 | |
| **Cancer site** |  |  |  |  |  |  | |
| Breast |  |  |  |  |  |  | |
| No | Ref. |  | Ref. |  | Ref. |  | |
| Yes | 0.80 (0.77-0.83) | <0.001 | 0.43 (0.21-0.89) | 0.022 | 0.61 (0.56-0.66) | <0.001 | |
| Prostate |  |  |  |  |  |  | |
| No | Ref. |  | Ref. |  | Ref. |  | |
| Yes | 0.91 (0.88-0.95) | <0.001 | 0.28 (0.13-0.61) | 0.001 | 0.66 (0.62-0.71) | <0.001 | |
| Lung and broncus |  |  |  |  |  |  | |
| No | Ref. |  | Ref. |  | Ref. |  | |
| Yes | 1.01 (0.98-1.03) | 0.667 | 0.65 (0.47-0.92) | 0.014 | 0.83 (0.80-0.86) | <0.001 | |
| Colon and rectum |  |  |  |  |  |  | |
| No | Ref. |  | Ref. |  | Ref. |  | |
| Yes | 0.96 (0.93-0.99) | 0.003 | 0.48 (0.29-0.77) | 0.003 | 1.00 (0.96-1.05) | 0.855 | |
| Melanoma of the skin |  |  |  |  |  |  | |
| No | Ref. |  | Ref. |  | Ref. |  | |
| Yes | 1.07 (0.91-1.25) | 0.413 | 0.00 (0.00-0.00) | <0.001 | 1.00 (0.78-1.30) | 0.972 | |
| Urinary bladder |  |  |  |  |  |  | |
| No | Ref. |  | Ref. |  | Ref. |  | |
| Yes | 0.84 (0.80-0.88) | <0.001 | 1.12 (0.62-2.04) | 0.699 | 1.65 (1.54-1.77) | <0.001 | |
| Non-hodgkin lymphoma |  |  |  |  |  |  | |
| No | Ref. |  | Ref. |  | Ref. |  | |
| Yes | 0.79 (0.76-0.83) | <0.001 | 1.30 (0.88-1.92) | 0.191 | 0.84 (0.78-0.90) | <0.001 | |
| Kidney and renal pelvis |  |  |  |  |  |  | |
| No | Ref. |  | Ref. |  | Ref. |  | |
| Yes | 1.53 (1.47-1.59) | <0.001 | 1.53 (0.97-2.43) | 0.070 | 2.77 (2.62-2.92) | <0.001 | |
| Corpus uteri |  |  |  |  |  |  | |
| No | Ref. |  | Ref. |  | Ref. |  | |
| Yes | 1.35 (1.27-1.43) | <0.001 | 0.83 (0.37-1.86) | 0.652 | 1.32 (1.20-1.46) | <0.001 | |
| Leukemia |  |  |  |  |  |  | |
| No | Ref. |  | Ref. |  | Ref. |  | |
| Yes | 0.76 (0.72-0.79) | <0.001 | 1.74 (1.25-2.41) | 0.001 | 0.79 (0.73-0.84) | <0.001 | |
| Pancreas |  |  |  |  |  |  | |
| No | Ref. |  | Ref. |  | Ref. |  | |
| Yes | 1.23 (1.18-1.28) | <0.001 | 0.52 (0.18-1.48) | 0.220 | 1.04 (0.98-1.11) | 0.231 | |
| Thyroid |  |  |  |  |  |  | |
| No | Ref. |  | Ref. |  | Ref. |  | |
| Yes | 1.03 (0.95-1.11) | 0.489 | 0.22 (0.03-1.53) | 0.125 | 0.76 (0.66-0.88) | <0.001 | |
| **Hospital characteristics** |  |  |  |  |  |  | |
| **Hospital bed size** |  |  |  |  |  |  | |
| Small | Ref. |  | Ref. |  | Ref. |  | |
| Medium | 1.04 (1.00-1.07) | 0.048 | 1.17 (0.73-1.88) | 0.515 | 1.05 (0.99-1.10) | 0.097 | |
| Large | 1.04 (1.00-1.07) | 0.027 | 1.20 (0.79-1.84) | 0.390 | 1.00 (0.95-1.05) | 0.937 | |
| **Region of hospital** |  |  |  |  |  |  | |
| Northeast | Ref. |  | Ref. |  | Ref. |  | |
| Midwest | 1.05 (1.02-1.09) | 0.001 | 2.92 (2.06-4.13) | <0.001 | 1.33 (1.26-1.41) | <0.001 | |
| South | 1.07 (1.03-1.10) | <0.001 | 1.65 (1.18-2.29) | 0.003 | 1.14 (1.08-1.20) | <0.001 | |
| West | 0.85 (0.82-0.87) | <0.001 | 2.31 (1.61-3.30) | <0.001 | 1.06 (1.00-1.12) | 0.061 | |
| **Location/teaching status of hospital** |  |  |  |  |  |  | |
| Rural | Ref. |  | Ref. |  | Ref. |  | |
| Urban non-teaching | 1.16 (1.10-1.23) | <0.001 | 2.23 (0.97-5.13) | 0.060 | 1.33 (1.23-1.45) | <0.001 | |
| Urban teaching | 1.14 (1.09-1.20) | <0.001 | 2.29 (1.06-4.94) | 0.034 | 1.28 (1.19-1.38) | <0.001 | |

**Supplementary Table S4. Predicted probabilities and standard errors of having overall, primary, secondary, and other hypertension by age, sex, race groups, and cancer type among hospitalized patients with cancer: results from multinomial logistic regression**

| **Cancer** | **Age** | | | | **Sex** | | **Race/Ethnicity** | | | | |
| --- | --- | --- | --- | --- | --- | --- | --- | --- | --- | --- | --- |
|  | **18-54** | **55-64** | **65-74** | **75+** | **Male** | **Female** | **White** | **Black** | **Hispanic** | **Asian** | **Other** |
| *Overall hypertension* |  |  |  |  |  |  |  |  |  |  |  |
| All cancer | 36.45% (0.002) | 55.65% (0.002) | 62.13% (0.002) | 69.33% (0.002) | 57.37% (0.002) | 56.09% (0.001) | 54.34% (0.001) | 68.73% (0.002) | 57.86% (0.003) | 58.04% (0.005) | 55.79% (0.004) |
| Breast | 26.58% (0.004) | 44.74% (0.005) | 51.45% (0.005) | 59.05% (0.005) | 42.36% (0.004) | 41.24% (0.004) | 38.27% (0.004) | 53.07% (0.005) | 41.63% (0.005) | 41.85% (0.007) | 39.78% (0.005) |
| Prostate | 33.34% (0.004) | 52.79% (0.004) | 59.38% (0.004) | 66.40% (0.004) | 54.60% (0.004) | N/A | 51.71% (0.004) | 66.81% (0.004) | 55.33% (0.005) | 55.58% (0.007) | 53.43% (0.005) |
| Lung/Bronchus | 39.21% (0.004) | 58.39% (0.003) | 64.70% (0.003) | 71.59% (0.003) | 63.60% (0.003) | 62.32% (0.003) | 60.86% (0.003) | 74.57% (0.003) | 64.33% (0.003) | 64.49% (0.005) | 62.28% (0.004) |
| Colon/Rectum | 36.13% (0.003) | 55.38% (0.003) | 61.88% (0.003) | 69.06% (0.003) | 57.68% (0.002) | 56.43% (0.002) | 54.81% (0.002) | 68.97% (0.003) | 58.28% (0.003) | 58.46% (0.006) | 56.26% (0.004) |
| Melanoma | 34.51% (0.016) | 53.84% (0.018) | 60.48% (0.017) | 67.92% (0.016) | 56.58% (0.016) | 55.27% (0.016) | 55.41% (0.016) | 69.75% (0.014) | 58.94% (0.016) | 59.10% (0.016) | 56.84% (0.016) |
| Urinary bladder | 40.76% (0.006) | 59.59% (0.006) | 65.96% (0.005) | 73.50% (0.005) | 66.75% (0.005) | 65.10% (0.005) | 64.78% (0.005) | 78.54% (0.004) | 68.37% (0.005) | 68.45% (0.007) | 65.95% (0.006) |
| Non- Hodgkin Lymphoma | 33.64% (0.005) | 52.62% (0.005) | 59.31% (0.005) | 67.05% (0.005) | 54.90% (0.004) | 53.47% (0.004) | 52.17% (0.004) | 67.01% (0.004) | 55.81% (0.005) | 55.96% (0.006) | 53.56% (0.006) |
| Kidney/Renal pelvis | 49.15% (0.005) | 68.24% (0.004) | 74.02% (0.004) | 80.37% (0.003) | 67.77% (0.004) | 66.35% (0.004) | 65.28% (0.004) | 78.57% (0.003) | 68.71% (0.004) | 68.82% (0.006) | 66.54% (0.005) |
| Corpus uteri | 42.15% (0.007) | 61.95% (0.006) | 68.12% (0.006) | 74.53% (0.006) | N/A | 62.65% (0.006) | 59.69% (0.006) | 73.52% (0.005) | 63.13% (0.006) | 63.34% (0.008) | 61.24% (0.007) |
| Leukemia | 33.42% (0.004) | 52.20% (0.005) | 58.86% (0.005) | 66.64% (0.005) | 51.67% (0.004) | 50.25% (0.004) | 48.76% (0.004) | 63.45% (0.005) | 52.31% (0.005) | 52.47% (0.006) | 50.09% (0.005) |
| Pancreas | 40.33% (0.005) | 59.84% (0.005) | 66.10% (0.004) | 72.81% (0.004) | 64.05% (0.004) | 62.85% (0.004) | 60.99% (0.004) | 74.61% (0.004) | 64.41% (0.005) | 64.59% (0.006) | 62.45% (0.005) |
| Thyroid | 30.47% (0.007) | 49.60% (0.009) | 56.32% (0.009) | 63.67% (0.008) | 43.71% (0.008) | 42.67% (0.008) | 41.01% (0.008) | 55.69% (0.008) | 44.35% (0.008) | 44.58% (0.009) | 42.56% (0.008) |
| *Primary hypertension* |  |  |  |  |  |  |  |  |  |  |  |
| All cancer | 28.08% (0.002) | 43.50% (0.002) | 47.96% (0.002) | 49.99% (0.002) | 42.25% (0.001) | 42.76% (0.001) | 41.17% (0.001) | 48.06% (0.002) | 42.91% (0.003) | 43.32% (0.005) | 42.91% (0.005) |
| Breast | 22.57% (0.003) | 38.19% (0.005) | 43.47% (0.005) | 47.38% (0.005) | 33.99% (0.004) | 34.01% (0.004) | 31.99% (0.004) | 41.93% (0.005) | 34.26% (0.005) | 34.61% (0.006) | 33.61% (0.006) |
| Prostate | 29.50% (0.004) | 46.73% (0.004) | 52.02% (0.004) | 55.47% (0.004) | 47.48% (0.004) | N/A | 45.51% (0.004) | 55.59% (0.004) | 48.01% (0.005) | 48.41% (0.007) | 47.41% (0.007) |
| Lung/Bronchus | 29.53% (0.003) | 44.71% (0.003) | 49.00% (0.003) | 50.93% (0.003) | 46.05% (0.003) | 46.66% (0.002) | 45.30% (0.002) | 51.39% (0.003) | 46.90% (0.004) | 47.32% (0.005) | 47.03% (0.006) |
| Colon/Rectum | 27.88% (0.003) | 43.42% (0.003) | 47.98% (0.003) | 50.27% (0.003) | 42.40% (0.002) | 42.89% (0.002) | 41.39% (0.002) | 48.35% (0.003) | 43.14% (0.003) | 43.55% (0.005) | 43.11% (0.006) |
| Melanoma | 26.98% (0.015) | 42.50% (0.017) | 47.10% (0.017) | 49.39% (0.018) | 42.00% (0.016) | 42.52% (0.016) | 41.75% (0.016) | 47.97% (0.018) | 43.35% (0.017) | 43.79% (0.017) | 43.49% (0.018) |
| Urinary bladder | 25.23% (0.005) | 38.12% (0.005) | 41.39% (0.005) | 41.25% (0.005) | 38.74% (0.005) | 40.05% (0.005) | 38.69% (0.005) | 40.41% (0.006) | 39.26% (0.006) | 39.76% (0.007) | 40.41% (0.008) |
| Non- Hodgkin Lymphoma | 24.06% (0.004) | 38.58% (0.005) | 42.96% (0.004) | 45.03% (0.005) | 37.44% (0.004) | 37.93% (0.004) | 36.53% (0.004) | 42.95% (0.005) | 38.13% (0.005) | 38.53% (0.006) | 38.20% (0.006) |
| Kidney/Renal pelvis | 34.88% (0.004) | 48.73% (0.004) | 51.60% (0.004) | 50.16% (0.004) | 45.24% (0.004) | 46.58% (0.004) | 45.19% (0.004) | 47.56% (0.005) | 45.93% (0.005) | 46.44% (0.006) | 46.99% (0.008) |
| Corpus uteri | 35.35% (0.006) | 52.13% (0.006) | 56.64% (0.006) | 58.57% (0.006) | N/A | 51.32% (0.006) | 49.63% (0.006) | 57.10% (0.006) | 51.57% (0.006) | 51.99% (0.008) | 51.44% (0.008) |
| Leukemia | 23.05% (0.004) | 37.20% (0.005) | 41.53% (0.005) | 43.66% (0.005) | 33.98% (0.004) | 34.37% (0.004) | 32.91% (0.004) | 39.65% (0.005) | 34.53% (0.005) | 34.89% (0.006) | 34.48% (0.007) |
| Pancreas | 32.17% (0.004) | 48.17% (0.005) | 52.57% (0.004) | 54.39% (0.005) | 48.72% (0.004) | 49.38% (0.004) | 47.79% (0.004) | 54.01% (0.005) | 49.44% (0.005) | 49.88% (0.006) | 49.57% (0.007) |
| Thyroid | 26.49% (0.007) | 43.22% (0.009) | 48.55% (0.009) | 52.18% (0.010) | 36.50% (0.008) | 36.50% (0.008) | 35.03% (0.008) | 44.93% (0.009) | 37.32% (0.008) | 37.68% (0.009) | 36.71% (0.009) |
| *Secondary hypertension* |  |  |  |  |  |  |  |  |  |  |  |
| All cancer | 0.15% (0.000) | 0.11%  (0.000) | 0.10% (0.000) | 0.12%  (0.000) | 0.11%  (0.000) | 0.11%  (0.000) | 0.11%  (0.000) | 0.14%  (0.000) | 0.13%  (0.000) | 0.14%  (0.000) | 0.11%  (0.000) |
| Breast | 0.07% (0.000) | 0.05%  (0.000) | 0.05% (0.000) | 0.06%  (0.000) | 0.06%  (0.000) | 0.06%  (0.000) | 0.05%  (0.000) | 0.08%  (0.000) | 0.06%  (0.000) | 0.07%  (0.000) | 0.05%  (0.000) |
| Prostate | 0.04% (0.000) | 0.03%  (0.000) | 0.03%  (0.000) | 0.04%  (0.000) | 0.03%  (0.000) | N/A | 0.03%  (0.000) | 0.04%  (0.000) | 0.04%  (0.000) | 0.04%  (0.000) | 0.03%  (0.000) |
| Lung/Bronchus | 0.12% (0.000) | 0.09%  (0.000) | 0.07%  (0.000) | 0.09%  (0.000) | 0.08%  (0.000) | 0.08%  (0.000) | 0.08%  (0.000) | 0.10%  (0.000) | 0.09%  (0.000) | 0.10%  (0.000) | 0.08%  (0.000) |
| Colon/Rectum | 0.07% (0.000) | 0.06%  (0.000) | 0.05%  (0.000) | 0.06%  (0.000) | 0.06%  (0.000) | 0.06%  (0.000) | 0.05%  (0.000) | 0.07%  (0.000) | 0.06%  (0.000) | 0.07%  (0.000) | 0.05%  (0.000) |
| Melanoma | 0.00% (0.000) | 0.00%  (0.000) | 0.00%  (0.000) | 0.00%  (0.000) | 0.00%  (0.000) | 0.00%  (0.000) | 0.00%  (0.000) | 0.00%  (0.000) | 0.00%  (0.000) | 0.00%  (0.000) | 0.00%  (0.000) |
| Urinary bladder | 0.18% (0.001) | 0.13%  (0.000) | 0.11%  (0.000) | 0.13%  (0.000) | 0.12%  (0.000) | 0.13%  (0.000) | 0.12%  (0.000) | 0.14%  (0.000) | 0.14%  (0.000) | 0.15%  (0.001) | 0.12%  (0.000) |
| Non- Hodgkin Lymphoma | 0.32% (0.001) | 0.25%  (0.000) | 0.22%  (0.000) | 0.26%  (0.001) | 0.25%  (0.000) | 0.25%  (0.000) | 0.23%  (0.000) | 0.31%  (0.001) | 0.28%  (0.001) | 0.30%  (0.001) | 0.23%  (0.001) |
| Kidney/Renal pelvis | 0.18% (0.000) | 0.12%  (0.000) | 0.10%  (0.000) | 0.11%  (0.000) | 0.12%  (0.000) | 0.12%  (0.000) | 0.12%  (0.000) | 0.14%  (0.000) | 0.13%  (0.000) | 0.15%  (0.000) | 0.11%  (0.000) |
| Corpus uteri | 0.11% (0.000) | 0.08%  (0.000) | 0.07%  (0.000) | 0.08%  (0.000) | N/A | 0.08%  (0.000) | 0.08%  (0.000) | 0.10%  (0.000) | 0.09%  (0.000) | 0.10%  (0.000) | 0.07%  (0.000) |
| Leukemia | 0.46% (0.001) | 0.36%  (0.001) | 0.31%  (0.001) | 0.38%  (0.001) | 0.37%  (0.001) | 0.37%  (0.001) | 0.34%  (0.001) | 0.47%  (0.001) | 0.41%  (0.001) | 0.45%  (0.001) | 0.34%  (0.001) |
| Pancreas | 0.08% (0.000) | 0.06%  (0.000) | 0.05%  (0.000) | 0.06%  (0.000) | 0.06%  (0.000) | 0.06%  (0.000) | 0.05%  (0.000) | 0.07%  (0.000) | 0.06%  (0.000) | 0.07%  (0.000) | 0.05%  (0.000) |
| Thyroid | 0.03% (0.000) | 0.03%  (0.000) | 0.02%  (0.000) | 0.03%  (0.000) | 0.03%  (0.000) | 0.03%  (0.000) | 0.03%  (0.000) | 0.04%  (0.000) | 0.03%  (0.000) | 0.03%  (0.000) | 0.03%  (0.000) |
| *Other hypertension* |  |  |  |  |  |  |  |  |  |  |  |
| All cancer | 8.22% (0.001) | 12.04% (0.001) | 14.07% (0.001) | 19.22% (0.002) | 15.00% (0.001) | 13.21% (0.001) | 13.06% (0.001) | 20.53% (0.002) | 14.83% (0.002) | 14.58% (0.003) | 12.78% (0.005) |
| Breast | 3.94% (0.001) | 6.49%  (0.002) | 7.93%  (0.002) | 11.61% (0.003) | 8.32%  (0.002) | 7.18%  (0.002) | 6.24%  (0.002) | 11.06% (0.003) | 7.31%  (0.002) | 7.18%  (0.002) | 6.13%  (0.003) |
| Prostate | 3.80% (0.001) | 6.03%  (0.001) | 7.33%  (0.002) | 10.89% (0.003) | 7.08%  (0.002) | N/A | 6.17%  (0.001) | 11.18% (0.003) | 7.28%  (0.002) | 7.13%  (0.003) | 6.00%  (0.003) |
| Lung/Bronchus | 9.56% (0.002) | 13.60% (0.002) | 15.63% (0.002) | 20.57% (0.002) | 17.46% (0.002) | 15.58% (0.001) | 15.49% (0.001) | 23.08% (0.003) | 17.34% (0.002) | 17.07% (0.003) | 15.18% (0.005) |
| Colon/Rectum | 8.18% (0.001) | 11.90% (0.002) | 13.85% (0.002) | 18.73% (0.002) | 15.22% (0.001) | 13.49% (0.001) | 13.38% (0.001) | 20.55% (0.002) | 15.08% (0.002) | 14.84% (0.003) | 13.10% (0.005) |
| Melanoma | 7.53% (0.006) | 11.34% (0.009) | 13.38% (0.010) | 18.54% (0.014) | 14.58% (0.011) | 12.76% (0.009) | 13.66% (0.010) | 21.79% (0.016) | 15.59% (0.012) | 15.32% (0.012) | 13.35% (0.011) |
| Urinary bladder | 15.34% (0.003) | 21.34% (0.004) | 24.46% (0.004) | 32.13% (0.005) | 27.89% (0.004) | 24.92% (0.004) | 25.97% (0.004) | 37.99% (0.006) | 28.97% (0.005) | 28.54% (0.007) | 25.43% (0.009) |
| Non- Hodgkin Lymphoma | 9.26% (0.002) | 13.80% (0.003) | 16.13% (0.003) | 21.76% (0.004) | 17.22% (0.003) | 15.29% (0.003) | 15.41% (0.003) | 23.75% (0.004) | 17.40% (0.003) | 17.13% (0.004) | 15.13% (0.006) |
| Kidney/Renal pelvis | 14.10% (0.003) | 19.39% (0.003) | 22.32% (0.003) | 30.10% (0.004) | 22.41% (0.003) | 19.65% (0.003) | 19.98% (0.003) | 30.86% (0.005) | 22.64% (0.004) | 22.23% (0.006) | 19.44% (0.008) |
| Corpus uteri | 6.69% (0.002) | 9.74%  (0.003) | 11.41% (0.003) | 15.87% (0.005) | N/A | 11.25% (0.003) | 9.98%  (0.003) | 16.32% (0.005) | 11.47% (0.004) | 11.25% (0.004) | 9.72%  (0.005) |
| Leukemia | 9.91% (0.002) | 14.64% (0.003) | 17.02% (0.003) | 22.60% (0.004) | 17.32% (0.003) | 15.51% (0.003) | 15.50% (0.003) | 23.33% (0.004) | 17.37% (0.003) | 17.13% (0.004) | 15.27% (0.006) |
| Pancreas | 8.08% (0.002) | 11.61% (0.002) | 13.48% (0.003) | 18.36% (0.003) | 15.27% (0.003) | 13.41% (0.002) | 13.15% (0.002) | 20.53% (0.004) | 14.91% (0.003) | 14.65% (0.004) | 12.83% (0.005) |
| Thyroid | 3.94% (0.002) | 6.35%  (0.003) | 7.74%  (0.004) | 11.46% (0.006) | 7.19%  (0.004) | 6.14%  (0.003) | 5.95%  (0.003) | 10.72% (0.006) | 7.00%  (0.004) | 6.86%  (0.004) | 5.82%  (0.004) |

**Supplementary Table S5. Adjusted incidence rate ratio (IRR) of length of stay (days) among hospitalized patients with cancer: Results from negative binomial regression**

| **Variable** | **IRR (95% CI)** | **P** |
| --- | --- | --- |
| **Patient characteristics** |  |  |
| **Hypertension types** |  |  |
| Non-hypertension | Ref. |  |
| Primary hypertension | 1.00 (1.00-1.01) | 0.316 |
| Secondary hypertension | 1.28 (1.17-1.39) | <0.001 |
| Other hypertension | 1.15 (1.13-1.17) | <0.001 |
| **Age groups** |  |  |
| 18-54 | Ref. |  |
| 55-64 | 1.03 (1.02-1.05) | <0.001 |
| 65-74 | 1.01 (0.99-1.02) | 0.450 |
| 75+ | 1.00 (0.98-1.01) | 0.594 |
| **Sex** |  |  |
| Female | Ref. |  |
| Male | 1.03 (1.02-1.04) | <0.001 |
| **Race** |  |  |
| White | Ref. |  |
| Black | 1.16 (1.14-1.17) | <0.001 |
| Hispanic | 1.06 (1.05-1.08) | <0.001 |
| Asian/pacific islander | 1.04 (1.02-1.07) | 0.001 |
| Other | 1.08 (1.03-1.13) | 0.001 |
| **Income** |  |  |
| 0-25^th^ | Ref. |  |
| 26-50^th^ | 0.98 (0.97-0.99) | <0.001 |
| 51-75^th^ | 0.95 (0.94-0.96) | <0.001 |
| 76-100^th^ | 0.94 (0.92-0.95) | <0.001 |
| **Insurance** |  |  |
| Medicare | Ref. |  |
| Medicaid | 1.14 (1.12-1.16) | <0.001 |
| Private insurance | 0.93 (0.92-0.94) | <0.001 |
| Other | 1.01 (0.98-1.03) | 0.521 |
| **Elixhauser index** |  |  |
| **Comorbidities** | 1.01 (1.01-1.01) | <0.001 |
| Atrial fibrillation |  |  |
| No | Ref. |  |
| Yes | 1.23 (1.22-1.25) | <0.001 |
| Coronary artery disease |  |  |
| No | Ref. |  |
| Yes | 1.01 (1.00-1.02) | 0.029 |
| Cardiomegaly |  |  |
| No | Ref. |  |
| Yes | 1.21 (1.11-1.31) | <0.001 |
| Cardiomyopathy |  |  |
| No | Ref. |  |
| Yes | 1.10 (1.07-1.14) | <0.001 |
| Heart failure |  |  |
| No | Ref. |  |
| Yes | 1.15 (1.13-1.17) | <0.001 |
| Peripheral artery disease |  |  |
| No | Ref. |  |
| Yes | 1.01 (0.99-1.03) | 0.289 |
| Stroke |  |  |
| No | Ref. |  |
| Yes | 1.32 (1.30-1.35) | <0.001 |
| **Cancer treatments** |  |  |
| Cancer surgery |  |  |
| No | Ref. |  |
| Yes | 1.16 (1.14-1.18) | <0.001 |
| Radiation |  |  |
| No | Ref. |  |
| Yes | 1.58 (1.53-1.63) | <0.001 |
| Chemotherapy |  |  |
| No | Ref. |  |
| Yes | 2.33 (2.27-2.40) | <0.001 |
| **Cancer site** |  |  |
| Breast |  |  |
| No | Ref. |  |
| Yes | 0.55 (0.53-0.57) | <0.001 |
| Prostate |  |  |
| No | Ref. |  |
| Yes | 0.34 (0.33-0.36) | <0.001 |
| Lung and broncus |  |  |
| No | Ref. |  |
| Yes | 0.88 (0.87-0.89) | <0.001 |
| Colon and rectum |  |  |
| No | Ref. |  |
| Yes | 1.00 (0.98-1.01) | 0.731 |
| Melanoma of the skin |  |  |
| No | Ref. |  |
| Yes | 0.71 (0.64-0.79) | <0.001 |
| Urinary bladder |  |  |
| No | Ref. |  |
| Yes | 0.97 (0.95-0.99) | 0.003 |
| Non-hodgkin lymphoma |  |  |
| No | Ref. |  |
| Yes | 1.29 (1.26-1.32) | <0.001 |
| Kidney and renal pelvis |  |  |
| No | Ref. |  |
| Yes | 0.66 (0.64-0.67) | <0.001 |
| Corpus uteri |  |  |
| No | Ref. |  |
| Yes | 0.60 (0.58-0.63) | <0.001 |
| Leukemia |  |  |
| No | Ref. |  |
| Yes | 1.80 (1.74-1.87) | <0.001 |
| Pancreas |  |  |
| No | Ref. |  |
| Yes | 1.05 (1.03-1.07) | <0.001 |
| Thyroid |  |  |
| No | Ref. |  |
| Yes | 0.52 (0.49-0.55) | <0.001 |
| **Hospital characteristic** |  |  |
| **Bed size** |  |  |
| Small | Ref. |  |
| Medium | 1.00 (0.95-1.05) | 0.984 |
| Large | 1.07 (1.01-1.12) | 0.013 |
| **Region** |  |  |
| Northeast | Ref. |  |
| Midwest | 0.92 (0.89-0.95) | <0.001 |
| South | 0.98 (0.94-1.01) | 0.182 |
| West | 0.94 (0.91-0.98) | 0.001 |
| **Location/teaching status** |  |  |
| Rural | Ref. |  |
| Urban non-teaching | 1.10 (1.07-1.13) | <0.001 |
| Urban teaching | 1.17 (1.14-1.19) | <0.001 |

**Supplementary Table S6. Predicted length of stays (days) and standard errors in primary, secondary, and other hypertension by age, sex, race groups, and cancer site among hospitalized patients with cancer: Results from negative binomial regression**

|  | **Length of stay (standard errors)** | | | |
| --- | --- | --- | --- | --- |
|  | **Primary hypertension** | **Secondary hypertension** | **Other**  **hypertension** | **Non-**  **hypertension** |
| **All cancer** | 6.46 (0.03) | 8.22 (0.36) | 7.40 (0.05) | 6.43 (0.04) |
| **Age groups** |  |  |  |  |
| 18-54 | 6.73 (0.05) | 8.55 (0.37) | 7.70 (0.07) | 6.70 (0.05) |
| 55-64 | 6.35 (0.03) | 8.07 (0.35) | 7.27 (0.05) | 6.32 (0.04) |
| 65-74 | 6.30 (0.04) | 8.01 (0.35) | 7.22 (0.05) | 6.27 (0.04) |
| 75+ | 6.57 (0.05) | 8.35 (0.36) | 7.52 (0.05) | 6.54 (0.06) |
| **Sex** |  |  |  |  |
| Male | 6.51 (0.04) | 8.28 (0.36) | 7.46 (0.05) | 6.48 (0.04) |
| Female | 6.41 (0.04) | 8.15 (0.35) | 7.34 (0.05) | 6.38 (0.04) |
| **Race/Ethnicity** |  |  |  |  |
| White | 6.17 (0.03) | 7.85 (0.34) | 7.07 (0.04) | 6.14 (0.03) |
| Black | 7.32 (0.06) | 9.31 (0.41) | 8.39 (0.07) | 7.29 (0.07) |
| Hispanic | 6.94 (0.07) | 8.82 (0.39) | 7.95 (0.08) | 6.91 (0.07) |
| Asian | 6.62 (0.09) | 8.41 (0.38) | 7.58 (0.10) | 6.59 (0.09) |
| Other | 7.49 (0.18) | 9.52 (0.48) | 8.58 (0.20) | 7.46 (0.18) |
| **Cancer site** |  |  |  |  |
| Breast | 3.26 (0.07) | 4.15 (0.20) | 3.74 (0.07) | 3.25 (0.07) |
| Prostate | 2.22 (0.04) | 2.82 (0.13) | 2.54 (0.04) | 2.21 (0.04) |
| Lung/Bronchus | 6.12 (0.05) | 7.78 (0.34) | 7.01 (0.05) | 6.09 (0.05) |
| Colon/Rectum | 6.70 (0.04) | 8.52 (0.37) | 7.68 (0.05) | 6.67 (0.04) |
| Melanoma | 4.51 (0.25) | 0.00 (0.00) | 5.17 (0.27) | 4.49 (0.25) |
| Urinary bladder | 6.33 (0.07) | 8.06 (0.35) | 7.26 (0.07) | 6.31 (0.07) |
| Non-Hodgkin lymphoma | 10.38 (0.12) | 13.20 (0.59) | 11.89 (0.15) | 10.33 (0.12) |
| Kidney/Renal pelvis | 3.81 (0.04) | 4.85 (0.21) | 4.36 (0.05) | 3.79 (0.04) |
| Corpus uteri | 3.94 (0.08) | 5.01 (0.24) | 4.51 (0.10) | 3.92 (0.09) |
| Leukemia | 15.24 (0.29) | 19.38 (0.90) | 17.46 (0.33) | 15.17 (0.28) |
| Pancreas | 6.89 (0.07) | 8.76 (0.38) | 7.89 (0.08) | 6.86 (0.07) |
| Thyroid | 3.14 (0.08) | 3.99 (0.20) | 3.60 (0.10) | 3.12 (0.08) |
